# Novel Application of Eupatilin for Effectively Attenuating Cisplatin-Induced Auditory Hair Cell Death via Mitochondrial Apoptosis Pathway

**DOI:** 10.1155/2022/1090034

**Published:** 2022-01-17

**Authors:** Xiaochan Lu, Tingting Deng, Hongsong Dong, Jinghong Han, Yanping Yu, Deng Xiang, Guohui Nie, Bing Hu

**Affiliations:** Shenzhen Key Laboratory of Nanozymes and Translational Cancer Research, Department of Otolaryngology, and Shenzhen Institute of Translational Medicine, Shenzhen Second People's Hospital/The First Affiliated Hospital of Shenzhen University Health Science Center, Shenzhen 518035, China

## Abstract

Eupatilin (5,7-dihydroxy-3′,4′,6-trimethoxyflavone) is a pharmacologically active flavone that has been isolated from a variety of medicinal plants and possesses a number of pharmacological properties. This study evaluates the antioxidant and antiapoptotic effects of eupatilin on cisplatin-induced ototoxicity using *in vitro* and *in vivo* models including HEI-OC1 cells, cochlear hair cells, and zebrafish. Employing a CCK8 assay and Annexin V-FITC/PI double staining, we found that eupatilin significantly alleviated cisplatin-induced apoptosis and increased hair cell viability. The level of reactive oxygen species (ROS) was evaluated by CellROX green and MitoSOX Red staining. The results showed that eupatilin possesses antioxidant activity. MitoTracker Red staining indicated that eupatilin remarkably decreased mitochondrial damage. Furthermore, we demonstrated that eupatilin protects hair cells from cisplatin-induced damage. Mechanistic studies in cisplatin-induced HEI-OC1 cells revealed that eupatilin promoted Bcl-2 expression, downregulated Bax expression, reversed the increase in caspase-3 and PARP activity, and reduced the expression of phosphorylated p38 and JNK. Our data suggest a novel role for eupatilin as a protective agent against ototoxic drug-induced hair cell apoptosis by inhibiting ROS generation and modulating mitochondrial-related apoptosis.

## 1. Introduction

At least 700 million people worldwide suffer from sensorineural hearing loss caused by ototoxic antibiotics, noise, chemotherapy, and aging [1]. There are currently no pharmacological agents aimed at preventing or treating sensorineural hearing loss that have been approved by the Food and Drug Administration (FDA). Drug-induced hearing loss is defined as a hearing impairment whose onset occurs when therapeutic drugs damage the auditory system as a side effect. More than 600 drugs have been identified to possess ototoxic properties. Among them, aminoglycoside antibiotics and platinum-containing chemotherapy drugs are the most investigated. Cisplatin- (Cis-) induced hearing loss, mainly in the high-frequency range, is bilateral and permanent which adversely affects the quality of life of cancer patients. Chemotherapy causes hearing loss in 40-60% of cancer patients, and children show a greater risk for developing hearing loss after cisplatin treatment than adults [2–4]. To address this side effect, it is critical to identify agents that provide protective measures against cisplatin-induced ototoxicity.

The mechanism of cisplatin ototoxicity has not been fully elucidated, and multiple factors and substances may be involved. Many studies have reported that the pathogenesis of ototoxicity is attributed to apoptosis of auditory hair cells accompanied by oxidative stress [5]. Oxidative stress originates from the overproduction of reactive oxygen species (ROS) after ototoxic insult [6]. Excessive ROS in cochlear hair cells leads to an imbalance in the redox environment, triggers mitochondrial cytochrome c release, and activates the caspase-3-dependent apoptosis pathway. p38 mitogen-activated protein kinase (MAPK) and c-Jun N-terminal kinase (JNK) are major members of the MAPK family and play important roles in cell growth, stress, inflammation, and apoptosis [7–9]. Previous studies revealed that p38 MAPK and JNK are involved in cochlear cell apoptosis induced by noise, gentamycin, and other ototoxic drugs [10, 11]. The field has made progress in understanding the mechanisms underlying drug-induced hearing loss, but the lack of pharmacological interventions for this prevalent disease is still an urgent problem for researchers and clinicians. This necessitates the development of effective therapies to abrogate drug-induced hearing loss.

Eupatilin (5,7-dihydroxy-3′,4′,6-trimethoxyflavone) is the active ingredient of Stillen, a commercially available extract from *Artemisia asiatica* [12, 13]. The extract has been widely used in South Korea to treat patients with gastric mucosal ulcers [14]. Researchers have found that eupatilin increases the transcriptional activity and expression of peroxisome proliferator-activated receptor *α* (PPAR*α*) and was proposed to be a potent agonist of PPAR*α* [15]. Fenofibrate, another PPAR*α* agonist, protects auditory hair cells from gentamycin-induced toxicity by reducing ROS levels and upregulating antioxidant enzymes [16]. Numerous studies have demonstrated the beneficial biological activities of eupatilin including antioxidation, anti-inflammation, and neuroprotection [17–21]. Several studies of eupatilin suggested that it has antitumor properties due to its proapoptotic activity in cancers such as leukemia, osteosarcoma, glioma, and melanoma [22–25]. Lou et al. showed that eupatilin could inhibit IL-1*β*-induced apoptosis via sestrin 2-dependent autophagy in chondrocytes [26]. Furthermore, eupatilin has been shown to exert protective effects against cisplatin-induced renal damage by scavenging ROS [20, 27]. In contrast, eupatilin could induce apoptosis in renal cancer cells via ROS-mediated phosphorylation of p38 MAPK, ERK1/2, JNK, and inhibition of the AKT/PI3K signaling cascade [20]. It is attractive to explore the phenomenon and mechanism of eupatilin on cisplatin-mediated ototoxicity, as this agent is safe in clinical practice and easily extracted from natural herbs.

In the present study, we aim to determine whether eupatilin attenuates cisplatin-induced hair cell damage *in vitro* and *in vivo* and to characterize the mechanism underlying this action.

## 2. Materials and Methods

### 2.1. HEI-OC1 Cell Culture and Drug Treatments

The House Ear Institute-Organ of Corti 1 (HEI-OC1) cell line is a widely used auditory hair cell line derived from the cochlea of the immortomouse [28], and it was cultured in high-glucose Dulbecco's modified Eagle's medium (DMEM; AG29643096; Hyclone) supplemented with 10% fetal bovine serum (FBS; 10099-141; Gibco) and grown under 33°C and 10% CO_2_. The cells were trypsinized at 80% confluence using 0.25% trypsin/EDTA (25200056; Life Technologies). Eupatilin was purchased from MCE (HY-N0783; MCE) and is dissolved at a stock concentration of 50 mM in dimethyl sulfoxide (DMSO; SHBM2270; Sigma-Aldrich) and further diluted to the desired concentrations. HEI-OC1 cells were pretreated with varying concentrations of eupatilin for 2 h and then cotreated with the optimal concentration (30 *μ*M) of cisplatin for 24 h (479306; Sigma-Aldrich), which is dissolved at a stock concentration of 1 mM in H_2_O.

### 2.2. Zebrafish Husbandry and Drug Treatments

The transgenic zebrafish line Tg (Brn3C:EGFP) was kindly provided by Professor Huawei Li at Fudan University (Shanghai, China). Adult zebrafish and larvae were raised and maintained at 28.5°C in a circulating system. Developmental stages were evaluated as days postfertilization (dpf). After 2 h preincubation with eupatilin, the zebrafish larvae at 4 dpf were cotreated with 30 *μ*M cisplatin and 50 *μ*M eupatilin for 24 h.

### 2.3. Cochlear Explant Culture and Drug Treatments

All animal experiments were approved by the Institutional Animal Care and Use Committee of the Institute of Translational Medicine of Shenzhen Second People's Hospital. Cochlear organs were purchased from Guangdong Medical Laboratory Animal Center (China). Organs from C57BL/6 J mice were dissected on the second day after birth. Cochlear explants were cultured in Dulbecco's modified Eagle's medium/nutrient mixture F-12 (DMEM/F12; SH30023.01; Thermo Scientific), supplemented with B27 (17504044; Invitrogen) and ampicillin (P0781; Sigma-Aldrich) at 37°C and 5% CO_2_. In the experimental group, the cochlear explants were treated with 50 *μ*M eupatilin for 2 h prior to 30 *μ*M cisplatin exposure for 24 h. In the cisplatin-only group, the cochlear explants were incubated with 30 *μ*M cisplatin for 24 h. After drug treatment, the cochlear explants were allowed to recover in fresh medium for two days. All cochlear explants were then fixed in 4% paraformaldehyde for immunofluorescence staining.

### 2.4. Cell Viability

Cell viability was evaluated using a Cell Counting Kit-8 (CCK8) assay kit following the manufacturer's instructions (CK04; Dojindo Laboratories). HEI-OC1 cells were seeded at a density of 1 × 10^4^ cells/well in 96-well plates (3599; Corning) and grown overnight at 33°C and 10% CO_2_. The cells were then treated with different concentrations of cisplatin and/or eupatilin for 24 h. After incubation, 10 *μ*L of CCK8 substrate and 90 *μ*L of medium were added to each well and incubated for 2 h. The optical density (OD) was recorded by a microplate reader at 450 nm (Spark 10M; TECAN). The cell viability was calculated as follows: (ODexperiment − OD background)/(ODnegative − ODbackground) × 100.

### 2.5. Protein Extraction and Western Blot Analysis

HEI-OC1 cells were suspended in lysis buffer (P0013C, Beyotime) containing protease inhibitors (04693159001, Roche) and a phosphatase inhibitor (49068370001, Roche) and allowed to lyse on ice for 45 min. The mixture was centrifuged at 12,000 rpm and 4°C for 15 min. The supernatant was collected, and the protein concentration was quantified using the Pierce™ BCA Protein Assay Kit (23227, Thermo Scientific). Twenty milligrams of protein was separated by 12% SDS-PAGE and then transferred to PVDF membranes (IPVH00010, Millipore). The membranes were blocked for 1 h at 25°C in TBST containing 5% milk and incubated with primary antibodies overnight at 4°C. The primary antibodies used in this experiment were tubulin (ab6046; Abcam), Bcl-2 (D17C4) rabbit mAb (3498T; Cell Signaling Technology), Bax Polyclonal Antibody (50599-2-IG; Proteintech), caspase-3 antibody (9662S; Cell Signaling Technology), cleaved caspase-3 (Asp175) (5A1E) rabbit mAb (9664S; Cell Signaling Technology), p38 MAPK (D13E1) XP rabbit mAb (8690S; Cell Signaling Technology), phospho-p38 MAPK (Thr180/Tyr182) (D3F9) XP rabbit mAb (4511S; Cell Signaling Technology), SAPK/JNK antibody (9252T; Cell Signaling Technology), phospho-SAPK/JNK (Thr183/Tyr185) (81E11) rabbit mAb (4668T; Cell Signaling Technology), and PARP (46D11) rabbit mAb (9532S; Cell Signaling Technology). The membranes were washed and then incubated with horseradish peroxidase-conjugated secondary antibodies (goat antimouse IgG H&L (HRP) (ab6789; Abcam) or goat antirabbit IgG H&L (HRP) (ab6721; Abcam)) for 1 h at 25°C. Finally, the membrane was stained with the Immobilon Forte Western HRP Substrate kit (WBLUF0100; Millipore), and bands were detected using an Amersham Imager 680 (US).

### 2.6. ROS Detection

Cellular ROS levels were detected using CellROX Green Reagent (C10444; Life Technologies) and MitoSOX Red (1771410; Life Technologies) according to the manufacturer's instructions. HEI-OC1 cells were treated with the conditions indicated in the figure legends, washed with serum-free DMEM, and incubated with 5 *μ*M CellROX green for 30 min or 5 *μ*M MitoSOX Red for 10 min. The results were analyzed by a fluorescence microscope.

### 2.7. Immunofluorescence Staining

Cochlear explants or zebrafish larvae were fixed with 4% paraformaldehyde for 1 h, washed three times with PBS, and permeabilized with 1% Triton X-100 in PBS at 25°C for 10 min. Permeabilized samples were then blocked with 10% bovine serum albumin in PBS for 1 h, followed by incubation with the primary antibodies overnight at 4°C. The primary antibodies used in this experiment were myosin VIIa rabbit polyclonal antibody (25–6790; Proteus Biosciences) and GFP polyclonal antibody (A11122; Invitrogen). The samples were rinsed three times with PBS and incubated with the relevant secondary antibodies at 37°C for 1 h away from light. The secondary antibodies were goat antirabbit IgG (H+L) Highly Cross-Adsorbed Secondary Antibody, Alexa Fluor Plus 555 (A32732; Invitrogen), and Alexa Fluor 488-labeled donkey antirabbit antibody (A21206; Invitrogen). The nuclei were labeled with DAPI (D523; Dojindo Laboratories) for 10 min at room temperature, and the fluorescent signals were captured under a fluorescence microscope.

### 2.8. MitoTracker Red Assay

After cisplatin or eupatilin treatment, a 100 nM MitoTracker Red CMXRos (MB6064; Meilunbio) working solution was prepared for staining. HEI-OC1 cells with MitoTracker Red dye were incubated for 30 min at 37°C. After incubation, the cells were washed three times with prewarmed PBS, and the nuclei were stained with DAPI for 10 min at 25°C. Finally, the cells were analyzed under a fluorescence microscope.

### 2.9. Flow Cytometric Analysis of Apoptosis

After treatment with the indicated concentrations of cisplatin and/or eupatilin for 48 h, the cells were harvested and washed with prewarmed PBS, and then, the cells were stained with the Annexin V-Alexa Fluor 647/PI Kit (FXP023-050; KeyGEN BioTECH) following the manufacturer's instructions. The cells were immediately analyzed using flow cytometry (BD Accuri C6 Plus; US).

### 2.10. Statistical Analysis

All experiments were repeated at least three times, and statistical analyses were conducted using a two-tailed Student's *t*-test in Prism (GraphPad software 8.0). Differences were considered statistically significant at *p* < 0.05.

## 3. Results

### 3.1. Eupatilin Pretreatment Reduced the Cisplatin-Induced Cytotoxicity in HEI-OC1 Cells

**Figure 1:**
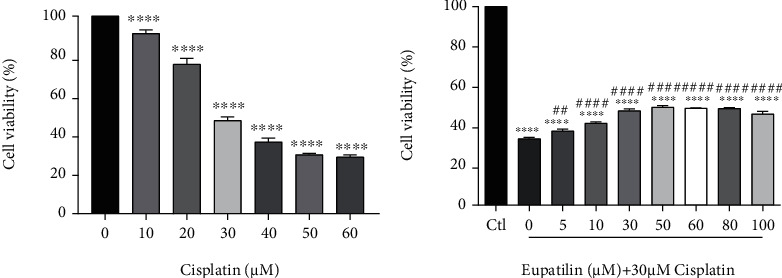
Eupatilin protected viability of HEI-OC1 cells upon cisplatin toxicity. (a) Effect of cisplatin on HEI-OC1 cells. HEI-OC1 cells were treated with or without various concentrations of cisplatin for 24 h. (b) Effect of eupatilin on cisplatin-induced viability in HEI-OC1 cells. Cells were pretreated with or without various concentrations of eupatilin (5-100 *μ*M) for 4 h followed by 30 *μ*M cotreatment for 24 h except those in the control (ctl) group. Cell viability was measured by CCK8 kit. Values were represented as the mean ± SEM from three independent experiments. ^∗∗∗∗^*p* < 0.0001 vs. the nontreated control group; ^##^*p* < 0.01 and ^####^*p* < 0.0001 vs. the group treated with 30 *μ*M cisplatin alone.

HEI-OC1 cells were derived from the immortalized cell line of the mouse organ of Corti, which is sensitive to ototoxic drugs including aminoglycosides and cisplatin. They have been commonly used as a cell model for studying ototoxicity. HEI-OC1 cells were treated with cisplatin at the dose and time conditions indicated in Figure 1(a) to establish an *in vitro* ototoxicity model. 30 *μ*M cisplatin was chosen as the optimal concentration for further studies. To determine whether eupatilin protected HEI-OC1 cells from cisplatin-induced damage, cells were pretreated with eupatilin concentrations of 0,5, 10, 30, 50, 60, 80, and 100 *μ*M for 4 h, and then cotreated with 30 *μ*M cisplatin for 24 h (Figure 1(b)). Using the CCK8 assay, we observed a significant protective effect of eupatilin at 30 *μ*M and 50 *μ*M, and a maximal protective effect at a concentration of 50 *μ*M. These results indicate that eupatilin can significantly protect against cisplatin-induced ototoxicity in HEI-OC1 cells.

### 3.2. Eupatilin Inhibited Cisplatin-Induced Apoptosis in HEI-OC1 Cells

**Figure 2:**
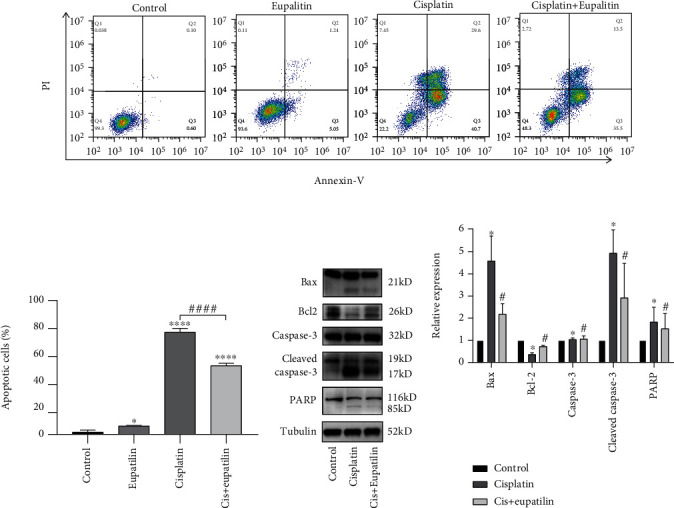
Eupatilin reduced cisplatin-induced apoptosis in HEI-OC1 cells. (a) HEI-OC1 cells were pretreated eupatilin for 4 h, followed by exposure of for 24 h except those in the control group. Cell apoptosis was determined by flow cytometry using Annexin V-Alexa Fluor 647/PI kit. (b) Quantitative analysis of apoptotic cells shown in (a). (c) Western blots for the expression of Bax, Bcl-2, caspase-3, cleaved caspase-3, and PARP in HEI-OC1 cells after pretreatment with eupatilin. (d) Quantitative analysis of the results shown in (c). Values are represented as the mean ± SEM from three independent experiments. ^∗^*p* < 0.05 and ^∗∗∗∗^*p* < 0.0001 vs. the nontreated control group; ^#^*p* < 0.05 and ^####^*p* < 0.0001 vs. the group treated with 30 *μ*M cisplatin alone.

Previous studies have shown that cisplatin induces apoptosis in HEI-OC1 cells via the mitochondrial apoptotic pathway [29]. To investigate whether the effects of eupatilin on HEI-OC1 cells were due to a reduction in apoptosis, Annexin V-FITC and PI double staining was analyzed by flow cytometry. Firstly, we detected the effect of solvent on the apoptosis of HEI-OC1 cells, including the nontreated control group, DMSO-treated control group, H_2_O-treated control group, and DMSO and H_2_O cotreated control group. The results showed that the solvent has no effect on cell apoptosis, so we used the nontreated control group in the following experiments (Supplementary Figure 1). Next, HEI-OC1 cells were divided into the following groups: nontreated control group, 50 *μ*M eupatilin treatment group, 30 *μ*M cisplatin treatment group, 30 *μ*M cisplatin and 50 *μ*M eupatilin cotreatment group. The cells were pretreated with 50 *μ*M eupatilin for 4 h. After 24 h in culture, the cells were double-stained with Annexin V-FITC and PI to analyze the percentage of apoptotic cells. Flow cytometry demonstrated that, after cisplatin treatment, cells had a significantly greater proportion of apoptotic cells (including late apoptotic cells in the upper right quadrant and early apoptotic cells in the lower right quadrant) than the nontreated control group (Figure 2(a)). The quantitative analysis showed that eupatilin cotreatment significantly reduced cisplatin-induced apoptosis in HEI-OC1 cells (Figure 2(b)). To explore these findings, we performed western blotting to examine whether HEI-OC1 cells underwent apoptosis following treatment with the indicated concentrations of cisplatin through the intrinsic pathway. As indicated in Figures 2(c) and 2(d), compared to the control, the expression of Bcl-2 was clearly downregulated by cisplatin in HEI-OC1 cells, while the proapoptotic proteins Bax and PARP were activated. Moreover, we observed that cisplatin treatment alone increased caspase-3 activity compared to the control group. In comparison, the expression of Bcl-2 was markedly attenuated, and the activity of Bax, PARP, and caspase-3 was significantly decreased following cisplatin exposure in the group pretreated with eupatilin compared to that in cells that were exposed to cisplatin alone. Taken together, these results implied that eupatilin inhibited the cisplatin-induced intrinsic apoptotic pathway in HEI-OC1 cells.

### 3.3. Eupatilin Reduced Cisplatin-Induced ROS Generation in HEI-OC1 Cells

**Figure 3:**
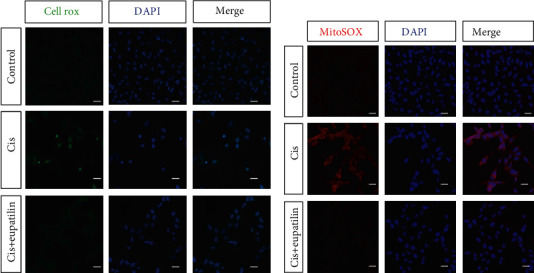
Eupatilin reduced ROS levels in cisplatin-induced HEI-OC1 cells. (a) The results of CellROX green staining for each group. (b) Effects of eupatilin on ROS production in cisplatin-damaged HEI-OC1 cells using MitoSOX Red staining. Scale bars equals 20 *μ*m.

Cisplatin treatment was reported to cause generation of ROS, which has long been recognized as an important activator of cisplatin-induced apoptosis. To examine whether eupatilin pretreatment inhibited cisplatin-induced accumulation of ROS, the levels of intracellular and mitochondrial ROS were measured by CellROX green and MitoSOX Red staining, respectively. As shown in Figure 3(a), there were almost no CellROX green positive cells in the control group. ROS production was significantly increased after treatment with cisplatin alone. However, pretreatment with eupatilin for 2 h inhibited ROS production induced by cisplatin exposure. In addition, HEI-OC1 cells exposed to cisplatin for 24 h showed an obvious increase in the red fluorescence of MitoSOX staining compared to that of the control group. In accordance with the CellROX green results, the MitoSOX red fluorescence was significantly reduced in the eupatilin and cisplatin cotreatment group, indicating that eupatilin protected cell death from cisplatin damage by reducing ROS production (Figure 3(b)).

### 3.4. Eupatilin Decreased Mitochondrial Damage in HEI-OC1 Cells

**Figure 4:**
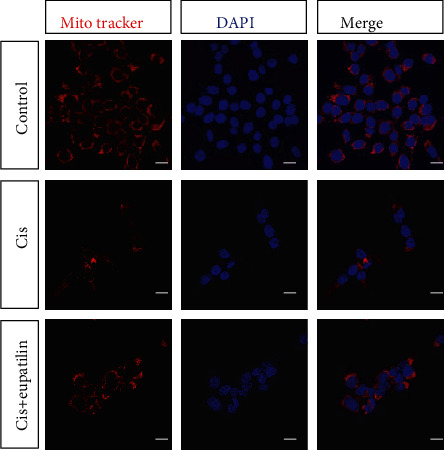
Eupatilin protected mitochondria from cisplatin toxicity in HEI-OC1 cells. MitoTracker Red staining showing different mitochondria changes following exposure to cisplatin with or without eupatilin. DAPI: 4′,6-diamidino-2-phenylindole. Scale bars equals 20 *μ*m.

We found that HEI-OC1 cells with cisplatin induced apoptosis through the generation of ROS and that eupatilin rescued cells from apoptosis through the intrinsic apoptotic pathway. Mitochondria are the main conductors of the intrinsic apoptotic pathway, and the loss of mitochondrial membrane potential (*ΔΨ*m) is an indicator of mitochondrial damage. We used MitoTracker Red staining to examine changes in mitochondrial membrane potential. As shown in Figure 4, the MitoTracker Red probe accumulated in the mitochondrial membrane and emitted a strong red fluorescent signal in the control group. The red fluorescence was significantly diminished in cells exposed to cisplatin only, indicating depolarization of the mitochondrial membrane. Eupatilin pretreatment recovered mitochondrial damage after cisplatin exposure, showing increased red fluorescence. These results suggest that eupatilin significantly inhibits cisplatin-induced apoptosis through the mitochondrial-related intrinsic apoptotic pathway.

### 3.5. Eupatilin Attenuated Cisplatin-Induced Cytotoxicity through the p38/JNK Pathway

**Figure 5:**
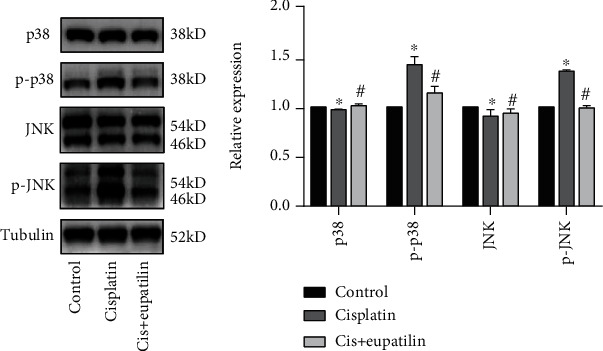
Eupatilin mediated HEI-OC1 cell death by affecting the p38/JNK signaling pathway. (a) Western blot results for p38, p-p38, JNK, and p-JNK in each group of HEI-OC1 cells. (b) Quantitative analysis of the results shown in (a). Values are represented as the mean ± SEM from three independent experiments. ^∗^*p* < 0.05 vs. the nontreated control group; ^#^*p* < 0.05 vs. the group treated with 30 *μ*M cisplatin alone.

The p38/JNK pathway contributes to an apoptotic response in stressed cells under various stimulus conditions, such as the accumulation of ROS. As shown in Figure 5(a), we evaluated cisplatin-induced changes in HEI-OC1 cells at the protein level. The phosphorylation levels of p38 and JNK were detected to determine whether eupatilin blocked the signaling pathway mediating the observed apoptotic responses. The results confirmed that the phosphorylation of p38 and JNK was increased in the cisplatin-treated group compared to that in the control group. However, eupatilin significantly reduced these changes after cisplatin exposure, suggesting that cisplatin induced apoptosis of HEI-OC1 via the p38/JNK pathway and eupatilin successfully attenuated cisplatin-induced cytotoxicity through the p38/JNK pathway (Figure 5(b)).

### 3.6. Eupatilin Protected against Cisplatin-Induced Hair Cell Loss in Zebrafish Lateral Line

**Figure 6:**
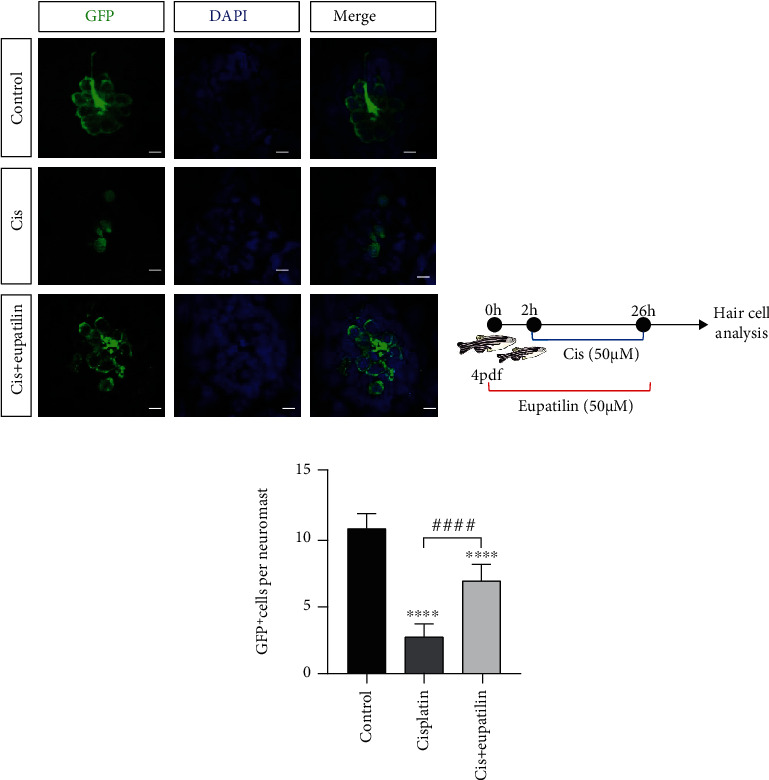
Eupatilin protected against cisplatin ototoxicity in transgenic zebrafish. (a) Zebrafish larvae at 4 dpf were exposed to cisplatin in the absence or presence of eupatilin for 24 h. The GFP fluorescence of hair cells was visualized under a fluorescence microscope. (b) Diagram of the assay for (a). (c) Quantification of the number of hair cells per neuromast after the different treatments represented as mean ± SEM. ^∗∗∗∗^*p* < 0.0001 vs. the nontreated control group; ^####^*p* < 0.0001 vs. the group treated with 30 *μ*M cisplatin alone. Scale bar equals 20 *μ*m.

Zebrafish offer unique advantages for studying defects in the inner ear. Lateral line hair cells are comparable to hair cells in the inner ear. The transgenic zebrafish line Tg (Brn3C:EGFP) expresses membrane-bound green fluorescent protein in hair cells, which is driven by the *brn3c* promoter. This allowed us to investigate the otoprotective effect of eupatilin *in vivo*. The effect of eupatilin solvent (DMSO) and eupatilin on zebrafish larvae has no difference compared to that on the nontreated control group (Supplementary Figure 2). Zebrafish larvae at 4 dpf were exposed to cisplatin (50 *μ*M) in the absence or presence of eupatilin (50 *μ*M) (Figures 6(a) and 6(b)). The results showed that the larvae treated with 50 *μ*M cisplatin for 24 h showed significant hair cell loss compared to the control larvae. However, there was a distinct protective effect on the larvae that were pretreated with 50 *μ*M eupatilin for 2 h, followed by cotreatment with cisplatin for 24 h (Figure 6(c)). These results revealed the protective effect of eupatilin against cisplatin-induced ototoxicity in a zebrafish model.

### 3.7. Eupatilin Protected Mouse Cochlear Hair Cells against Cisplatin-Induced Damage

**Figure 7:**
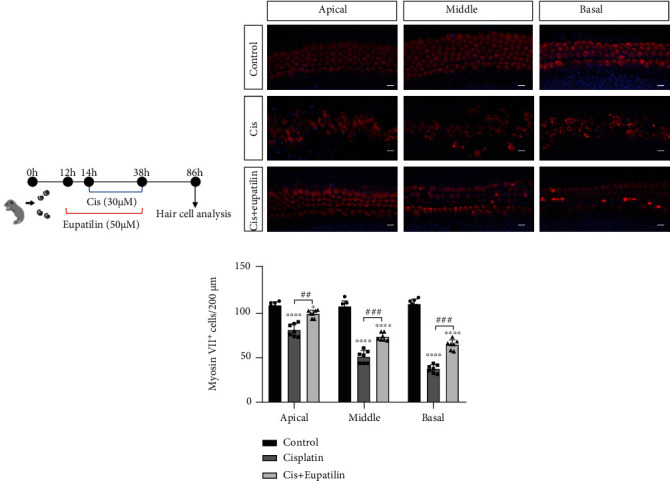
Effect of eupatilin on the cisplatin-induced apoptosis of cochlear hair cells. (a) Experimental design for (b) immunofluorescence staining with myosin VII (red) and DAPI (blue) in apical, middle, and basal turns of the cochlear from control, cisplatin-only, and cisplatin plus eupatilin groups. Scale bar equals 20 *μ*m. (c) Hair cells positive for myosin VII fluorescence were counted every 200 *μ*m along the apical, middle, and basal regions of cochlear explants from different groups. The data are presented as the mean ± SEM. ^∗^*p* < 0.05 and ^∗∗∗∗^*p* < 0.0001 vs. the nontreated control group; ^##^*p* < 0.01, ^###^*p* < 0.001, and ^####^*p* < 0.0001 vs. the group treated with the cisplatin group.

To evaluate the protective potential of eupatilin on cochlear hair cells, we first tested the effect of the eupatilin solvent group and the eupatilin-only group on cochlear hair cells. The results showed that the eupatilin solvent group and the eupatilin group were similar to the nontreated control group; no hair cell was damaged (Supplementary Figure 3). Next, cochlear explants were treated with cisplatin or eupatilin in the following groups: nontreated control group, 30 *μ*M cisplatin treatment group, 30 *μ*M cisplatin, and 50 *μ*M eupatilin cotreatment group. In the cotreatment group, cochlear explants were treated with eupatilin for 2 h prior to cisplatin exposure (Figure 7(a)). We visualized the survival of hair cells across three turns of the cochlear explants by myosin VIIa and DAPI immunostaining (Figure 7(b)). We quantified the number of hair cells on each turn of the basal, middle, and apical segments after each set of treatments (Figure 7(c)). The results showed that cisplatin treatment alone significantly reduced the number of hair cells compared to that of the untreated control group. In contrast, the cisplatin and eupatilin cotreatment groups displayed protective effects, reducing hair cell loss (Figure 7). Taken together, these results clearly indicate that eupatilin protects hair cells from cisplatin-induced cell death.

## 4. Discussion

Eupatilin is a pharmacologically active flavonoid mainly found in the genus *Artemisia*. It is known to possess antioxidant, anti-inflammatory, and antiapoptotic effects [30, 31]. In addition, studies on eupatilin have demonstrated that it also has anticancer, neuroprotective, antiallergic, and cardioprotective activities [32]. Eupatilin has been widely used to treat patients in South Korea with gastric mucosal ulcers [14]. However, the effects of eupatilin on ototoxicity-induced hearing loss and the potential mechanisms involved have not been explored. Here, we showed that eupatilin produced a protective effect against cisplatin-induced ototoxicity both *in vivo* and *in vitro*. In the CCK8 assay, we found that eupatilin protected HEI-OC1 cells from cisplatin-induced apoptosis, and the results were confirmed by Annexin V-FITC/PI staining and flow cytometry analysis. Our study is the first to report that eupatilin can be utilized as an otoprotective agent to protect against hair cell damage caused by cisplatin.

Cisplatin is approved by the FDA and is an effective drug for a broad range of cancers [33]. Cisplatin damages tumor cells by cross-linking with DNA and inducing apoptosis, but it also has off-target effects such as hepatotoxicity, nephrotoxicity, and ototoxicity [34]. Consequently, these irreversible toxicities reduce the potential options for cisplatin as a future treatment for relapse. To date, no FDA-approved drugs have been used to treat ototoxicity. Previous studies have shown that cisplatin induces apoptosis of hair cells through activation of the mitochondrial-mediated intrinsic apoptotic pathway, which is characterized by increased Bax/Bcl-2 proportion, decline in mitochondrial membrane potential, release of cytochrome c, activation of caspase-3, and cleavage of PARP [35]. In our *in vitro* model of cisplatin-induced toxicity in HEI-OC1 cells, we observed that cisplatin treatment markedly increased the activity of the proapoptotic protein Bax, downregulated the antiapoptotic protein Bcl-2, and improved the activities of caspase-3 and PARP. Eupatilin cotreatment effectively reversed these effects. These results confirmed the beneficial effect of eupatilin on HEI-OC1 apoptosis, suggesting that eupatilin stabilizes mitochondria and relieves the activation of mitochondria-mediated apoptosis, thereby protecting cells from cisplatin-induced apoptosis.

It has been demonstrated that mitochondria play a major role in ototoxic drug-induced ototoxicity, which is caused by excessive generation of ROS in hair cells [36, 37]. We analyzed the crosstalk between eupatilin, ROS, and mitochondria using a cellular CellROX green assay and the mitochondria-specific ROS indicator MitoSOX Red. Our results indicated that cisplatin increased the production of cellular ROS and mitochondria-specific ROS, leading to apoptotic characteristics in HEI-OC1 cells, consistent with previous findings. However, eupatilin treatment significantly decreased ROS production. In addition, we confirmed that eupatilin prevented cisplatin-induced mitochondrial damage using MitoTracker Red staining. Taken together, we observed that eupatilin protects against cisplatin-induced apoptosis by reducing ROS accumulation and decreasing the change in mitochondrial membrane potential. This suggests that eupatilin's antioxidant property might be the primary mechanism of its antiapoptotic effect on hair cells.

It is well known that JNK can be activated in response to oxidative stress. This acts as a signal of hair cell damage, and modulation of JNK has been proposed as a protective measure for the prevention of hearing loss [38]. Studies have demonstrated that p38 MAPK and JNK play critical roles in cochlear hair cell apoptosis induced by noise and ototoxic drugs [39]. Furthermore, Park et al. reported tha*t A. asiatica* and eupatilin cotreatment could rescue cisplatin-induced kidney damage by downregulating phosphorylated JNK and p38 protein levels. It has been reported that eupatilin also has a strong ability to induce apoptosis in renal cancer cells via ROS-mediated phosphorylation of p38 MAPK, JNK, and ERK1/2 [20]. Previous studies have shown that both cisplatin-induced apoptosis and eupatilin-mediated protection are associated with the p38 MAPK/JNK signaling pathway [40–42]. Thus, we investigated whether eupatilin exerted its protective effects against cisplatin through the p38 MAPK/JNK pathway. We found that pretreatment with eupatilin led to decreased phosphorylation of p38 MAPK and JNK after cisplatin administration, indicating that the antiapoptotic effect of eupatilin on cisplatin-induced ototoxicity might be partly attributed to the inhibition of phosphorylation of the p38 MAPK/JNK signaling pathway. Despite these studies, the underlying mechanisms through which eupatilin affects hair cell survival in response to ototoxic insults remain unclear. Next, we will employ appropriate pharmacological and genetic inhibition approaches to confirm whether hair cell protection by eupatilin is mediated by these signals.

## 5. Conclusion

**Figure 8:**
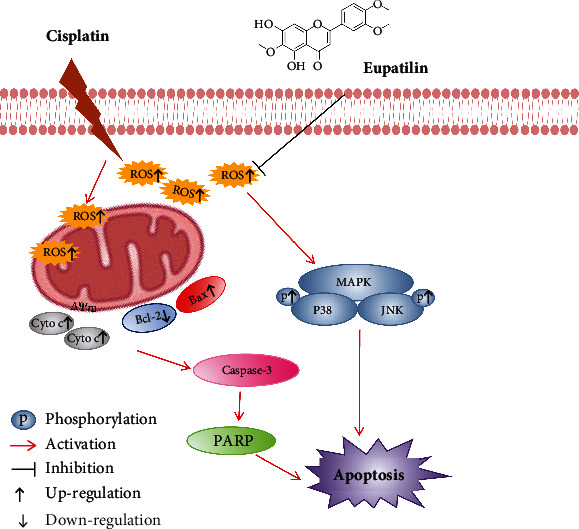
Schematic illustration of the possible mechanism of the protective effect of eupatilin against ototoxic drug-induced apoptosis. As confirmed, cisplatin treatment markedly induces the generation of ROS and triggers the mitochondrial apoptotic pathway. Eupatilin treatment could reduce the generation of ROS, increase the expression levels of antiapoptotic proteins, decrease the expression levels of proapoptotic proteins, and reduce the phosphorylation of p38 and JNK protein, suggesting that eupatilin protect hair cell death through the p38/JNK signaling pathway and finally ameliorate the oxidative stress-induced apoptosis.

In conclusion, we demonstrated for the first time that eupatilin could protect against cisplatin-induced ototoxicity *in vitro* (HEI-OC1 cells and isolated mouse cochlea) and *in vivo* (zebrafish larvae) by suppressing the generation of ROS and the mitochondrial-mediated apoptotic pathway, and the p38 MAPK/JNK pathway also participates in antiototoxicity (as summarized in Figure 8). Our present study provides solid evidence that eupatilin is promising as a potential medicine to protect patients against cisplatin-induced ototoxicity.

## Data Availability

The data that support the findings of this study are available from the corresponding author upon request.

## Supplementary Materials

Supplementary Materials Supplementary Figure 1: effect of solvent group on apoptosis of HEI-OC1 cells. HEI-OC1 cells were treated with the same concentration of DMSO or H_2_O as the experimental group for 24 h, and cell apoptosis was determined by flow cytometry using Annexin V and PI staining kits. Supplementary Figure 2: effect of eupatilin solvent (DMSO) and eupatilin on transgenic zebrafish. Zebrafish larvae at 4 dpf were exposed to DMSO and 50 *μ*M eupatilin for 24 h. The GFP fluorescence of hair cells was visualized under a fluorescence microscope. Scale bar equals 20 *μ*m. Supplementary Figure 3: effect of eupatilin solvent (DMSO) and eupatilin on cochlear hair cells. Immunofluorescence staining with myosin VII (red) and DAPI (blue) in the apical, middle, and basal turns of the cochlear from DMSO and eupatilin-only groups. Scale bar equals 20 *μ*m. Supplementary Figure 4: eupatilin protected against cisplatin ototoxicity in transgenic zebrafish. Zebrafish larvae at 4 dpf were exposed to 30 *μ*M cisplatin 2 h and then con-treated with 30 *μ*M cisplatin and 10 *μ*M eupatilin for 24 h. The GFP fluorescence of hair cells was visualized under a fluorescence microscope. Scale bar equals 20 *μ*m.
