# Supplementary material for: Novel Application of Eupatilin for Effectively Attenuating Cisplatin-Induced Auditory Hair Cell Death via Mitochondrial Apoptosis Pathway
Source: Oxid Med Cell Longev. 2022 Jan 17;2022:1090034. doi: 10.1155/2022/1090034 (PMC8786471; doi:10.1155/2022/1090034)
Supplement: Supplementary Materials — Supplementary Figure 1: effect of solvent group on apoptosis of HEI-OC1 cells. HEI-OC1 cells were treated with the same concentration of DMSO or H2O as the experimental group for 24 h, and cell apoptosis was determined by flow cytometry using Annexin V and PI staining kits. Supplementary Figure 2: effect of eupatilin solvent (DMSO) and eupatilin on transgenic zebrafish. Zebrafish larvae at 4 dpf were exposed to DMSO and 50 μM eupatilin for 24 h. The GFP fluorescence of hair cells was visualized under a fluorescence microscope. Scale bar equals 20 μm. Supplementary Figure 3: effect of eupatilin solvent (DMSO) and eupatilin on cochlear hair cells. Immunofluorescence staining with myosin VII (red) and DAPI (blue) in the apical, middle, and basal turns of the cochlear from DMSO and eupatilin-only groups. Scale bar equals 20 μm. Supplementary Figure 4: eupatilin protected against cisplatin ototoxicity in transgenic zebrafish. Zebrafish larvae at 4 dpf were exposed to 30 μM cisplatin 2 h and then con-treated with 30 μM cisplatin and 10 μM eupatilin for 24 h. The GFP fluorescence of hair cells was visualized under a fluorescence microscope. Scale bar equals 20 μm. [file 1090034.f1.docx]

**Novel Application of Eupatilin for effectively Attenuating Cisplatin-Induced Auditory Hair Cell Death via Mitochondrial Apoptosis Pathway**

**Xiaochan Lu,^1^ Tingting Deng,^1^ Hongsong Dong,^1^Jinghong Han, ^1^Yanping Yu,^1^Deng Xiang,^1^ Guohui Nie,^1^and Bing Hu^1^**


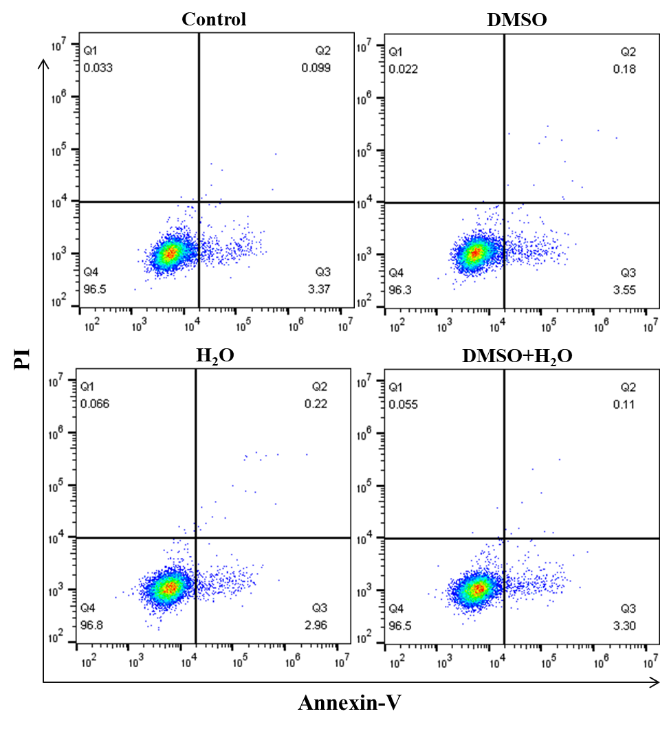


Supplementary Figure 1: Effect of solvent group on apoptosis of HEI-OC1 cells. HEI-OC1 cells were treated with the same concentration of DMSO or H_2_O as the experimental group for 24 h, and cells apoptosis was determined by flow cytometry using Annexin V and PI staining kits.


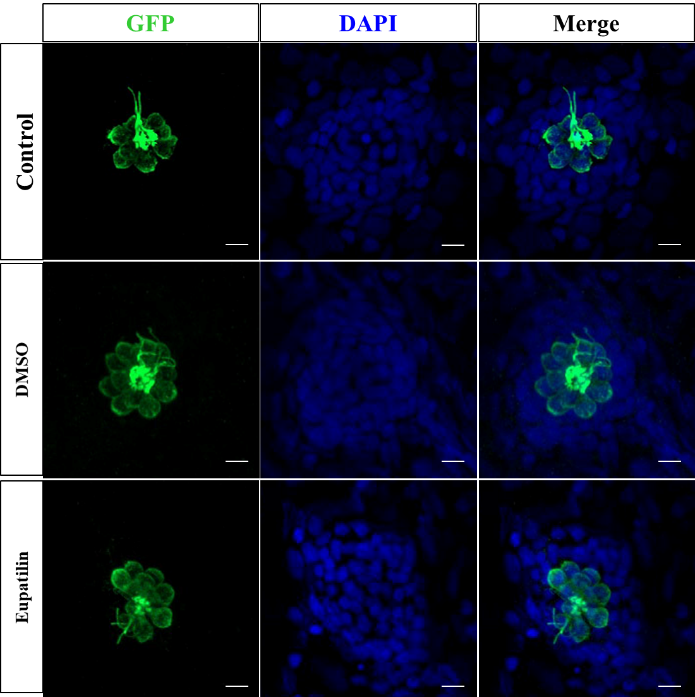


Supplementary Figure 2: Effect of eupatilin solvent(DMSO) and eupatilin on transgenic zebrafish. Zebrafish larvae at 4 dpf were exposed to DMSO and 50 μM eupatilin for 24 h. The GFP fluorescence of hair cells was visualized under a fluorescence microscope. Scale bar equals 20 μm.


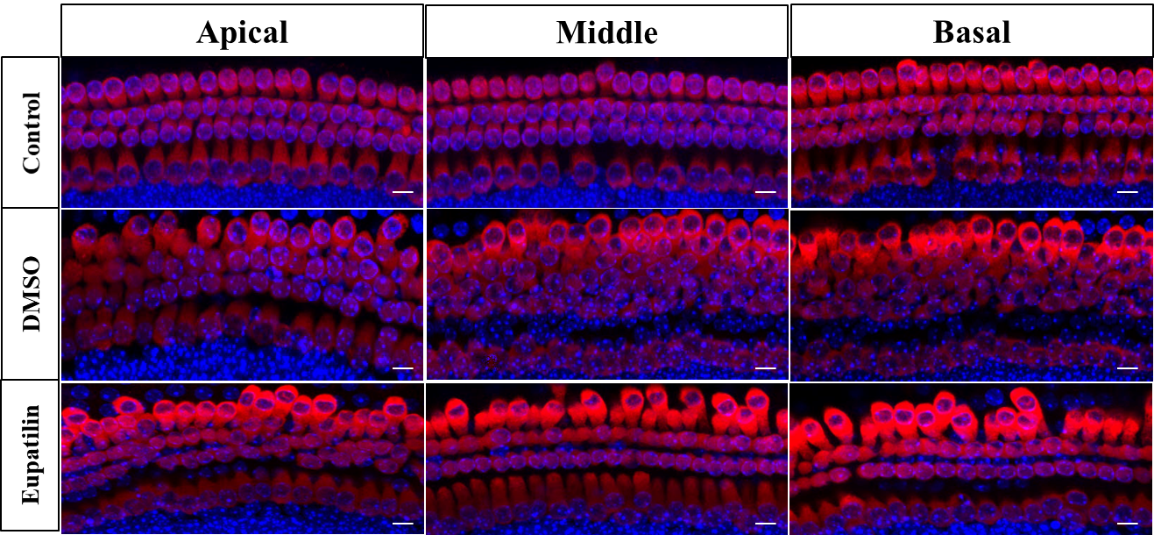


Supplementary Figure 3: Effect of eupatilin solvent(DMSO) and eupatilin on cochlear hair cells. Immunofluorescence staining with myosin VII(red) and DAPI(blue) in the apical, middle and basal turns of the cochlear from DMSO and eupatilin-only groups. Scale bar equals 20 μm.


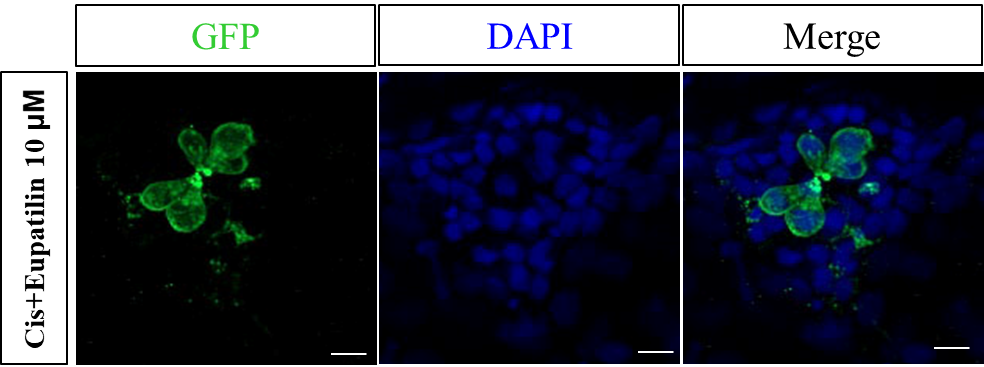


Supplementary Figure 4: Eupatilin protected against cisplatin ototoxicity in transgenic zebrafish. Zebrafsih larvae at 4 dpf were exposed to 30 μM cisplatin 2 h and then con-treated with 30 μM cisplatin and 10 μM eupatilin for 24 h. The GFP fluorescence of hair cells was visualized under a fluorescence microscope. Scale bar equals 20 μm.
